# A Rare Syndrome in a Rare Carcinoma: A Case of Spontaneous Tumor Lysis Syndrome in Small-Cell Liver Carcinoma

**DOI:** 10.7759/cureus.35455

**Published:** 2023-02-25

**Authors:** Deborah A Aina, Ashley Erwin, Elton Li, Rajendra Manam

**Affiliations:** 1 Internal Medicine, Ascension Providence Hospital, Southfield, USA; 2 Family Medicine, Ascension Providence Hospital, Southfield, USA; 3 Hematology and Oncology, Ascension Providence Hospital, Southfield, USA

**Keywords:** small-cell liver carcinoma, allopurinol, rasburicase, hyperuricemia, spontaneous tumor lysis syndrome

## Abstract

Spontaneous tumor lysis syndrome (STLS) is a rare, oncologic emergency, consisting of life-threatening acute renal failure, hyperuricemia, hyperkalemia, and hyperphosphatemia, prior to the initiation of cytotoxic therapy. Here, we describe a case of STLS in a patient with newly diagnosed small-cell liver carcinoma (SCLC). A 64-year-old female with no significant past medical history presented with a one-month history of jaundice, pruritus, pale stools, dark urine, and right upper quadrant pain. CT of the abdomen revealed a heterogeneous-enhancing intrahepatic mass. CT-guided biopsy of the mass revealed SCLC. At follow-up, labs were significant for potassium 6.4 mmol/L, phosphorus 9.4 mg/dL, uric acid 21.4 mg/dL, calcium 9.0 mg/dL, and creatinine 6.9 mg/dL. She was admitted and treated with aggressive fluid rehydration and rasburicase, with eventual improvement in renal function and normalization of electrolytes and uric acid levels. On the rare occasion that STLS occurs in solid tumors, lung, colorectal, and melanoma are most common, with liver metastases occurring in 65% of cases. Our patient’s SCLC was a primary liver malignancy with a large tumor burden, both of which may have predisposed her to develop STLS. Rasburicase is the first line in the treatment of acute tumor lysis syndrome as it quickly decreases uric acid. Identifying SCLC as a risk factor for STLS is key. Due to the high morbidity and mortality associated with this rare phenomenon, a prompt diagnosis is required.

## Introduction

Spontaneous tumor lysis syndrome (STLS) is a rare, oncologic emergency. It describes the development of life-threatening acute renal failure, hyperuricemia, hyperkalemia, and hyperphosphatemia, prior to the initiation of cytotoxic therapy. It is most commonly associated with hematologic malignancies. There are only 63 reported cases of STLS in solid tumors [1]. This is the first case of STLS in a patient with newly diagnosed small-cell liver carcinoma (SCLC).

## Case presentation

**Figure 1:**
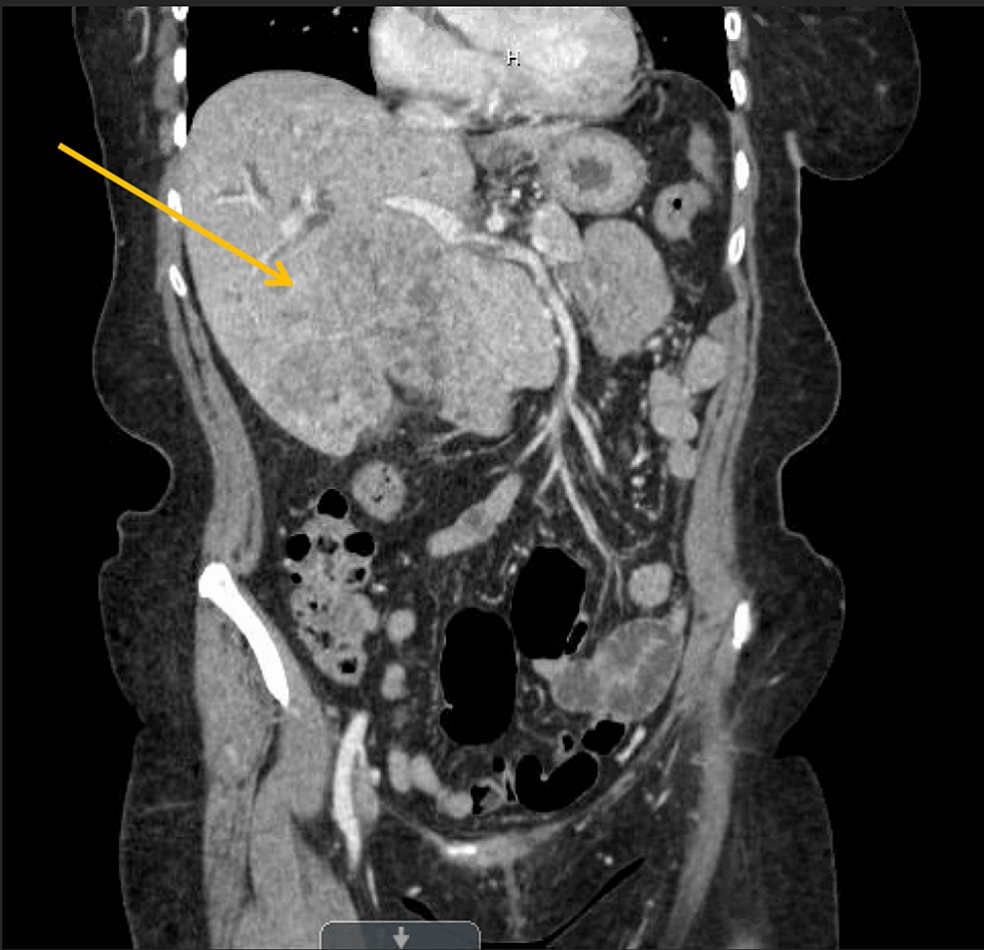
CT image of a 6.4 × 8.9 × 8.8 cm heterogenous-enhancing intrahepatic mass, with an invasion of the gallbladder, and intrahepatic biliary dilation.

**Figure 2:**
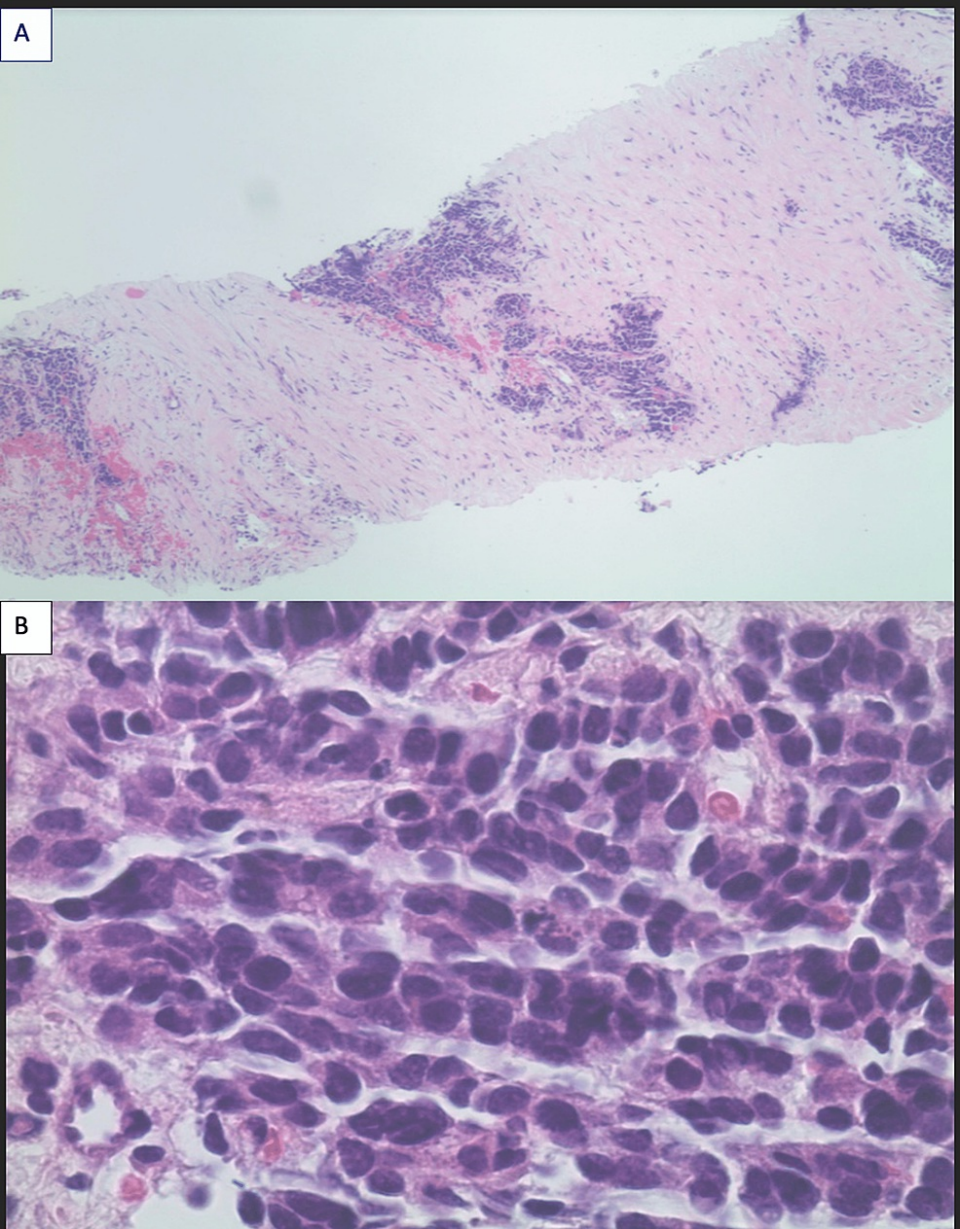
Hematoxylin and eosin stain of the small-cell liver carcinoma (A: 40×; B: 400×).

A 64-year-old female with no significant past medical history and no prior history of malignancy presented with a one-month history of jaundice, pruritus, pale stools, dark urine, fatigue, and right upper quadrant pain. CT of the abdomen revealed a large heterogenous-enhancing intrahepatic mass, with an invasion of the gallbladder, and intrahepatic biliary dilation (Figure 1). Initial labs revealed aspartate transaminase of 139 U/L, alanine transaminase of 106 U/L, total bilirubin of 4.8 mg/dL, and alkaline phosphatase of 650 U/L, indicating obstructive pathology. CT-guided biopsy results demonstrated small-cell carcinoma, suspicious for a primary hepatic malignancy (Figure 2). She underwent Y90 mapping and right portal vein embolization, with two percutaneous transhepatic cholangiogram drains placed on discharge. On follow-up, labs were significant for potassium 6.4 mmol/L, phosphorus 9.4 mg/dL, uric acid 21.4 mg/dL, calcium 9.0 mg/dL, and creatinine 6.9 mg/dL, with concern for STLS. She was readmitted and treated with aggressive fluid rehydration and rasburicase, with improvement in her renal function and normalization of her electrolytes and uric acid levels on discharge.

## Discussion

Here, we present the first case of STLS as a complication of SCLC. There are only 63 reported cases of STLS in solid tumors. Lung, colorectal, and melanoma are the most commonly reported, with liver metastases occurring in 65% of cases [1]. Our patient’s carcinoma was of liver primary malignancy with a large tumor burden, two things that may have predisposed her to develop STLS. Impaired uric acid metabolism and the systemic inflammatory response brought on by necrosis in a cancerous bulk are believed to contribute [2]. Other risk factors for the development of STLS include old age, high lactate dehydrogenase level, white blood cell count, and history of renal impairment [2]; though these factors are better associated with hematologic malignancies.

**Figure 3:**
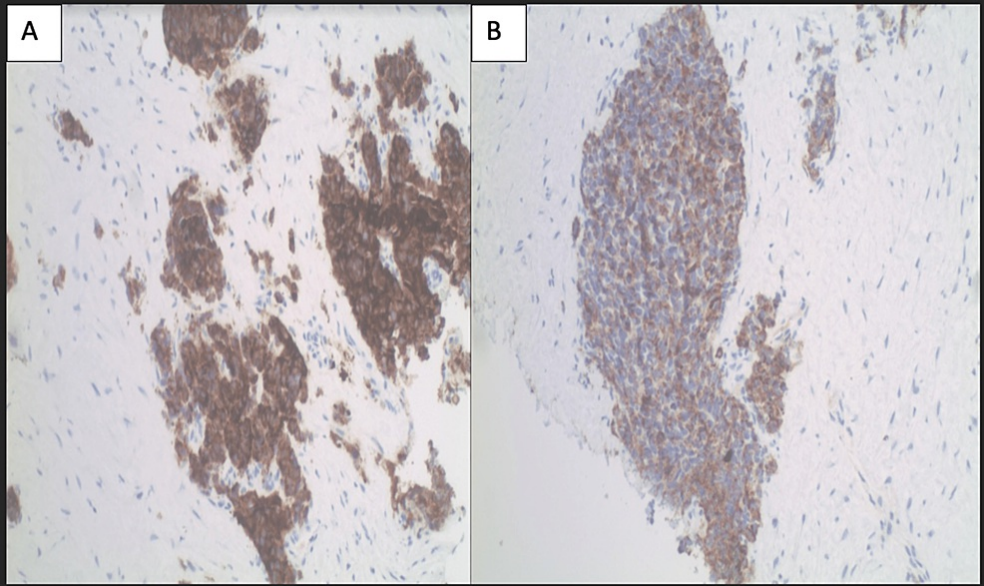
Immunohistochemical staining. The tumor is positive for synaptophysin 100× (A) and chromogranin 100× (B).

There have only been 12 reported cases of SCLC in the literature, therefore, information regarding this tumor’s clinical, pathological, and immunohistochemistry features is limited [3]. Histologically, our patient’s tumor showed typical pathological morphology of pulmonary small-cell lung carcinoma, though a CT of the chest was negative for metastatic disease. Immunohistochemically, the tumor was positive for synaptophysin, chromogranin, CD56, and BerEP-4, consistent with SCLC [4] (Figure 3).

In our case, our patient was diagnosed with STLS 15 days following the new diagnosis of SCLC prior to the initiation of chemotherapy. Her hyperkalemia, hyperphosphatemia, hyperuricemia, and acute kidney injury met the definition for tumor lysis syndrome (TLS) by the Cairo-Bishop laboratory definition: uric acid ≥8 mg/dL, potassium ≥6 mEq/dL, phosphorus ≥4.6 mg/dL; as well as the clinical definition: acute kidney injury >1.5× upper limit of patient’s baseline (0.8 mg/dL) in our patient [5]. Our patient’s calcium was within normal limits, though only two of the four laboratory criteria are required to make the diagnosis of laboratory TLS. Clinical TLS diagnosis is additionally made by the presence of acute kidney injury.

Once the diagnosis was made in our patient, she was treated with aggressive intravenous fluid rehydration, along with rasburicase. Rasburicase is the first line in the treatment of acute TLS as it decreases the uric acid level quickly. Renal replacement therapy with dialysis is recommended for refractory electrolyte abnormalities, along with severe oliguria and anuria [6]. Though our patient was oliguric, the decision was made for conservative management as she was not fluid-overloaded and her electrolytes showed improvement with fluids and medical management alone.

## Conclusions

This case introduces STLS as a complication of SCLC. Due to the high morbidity and mortality associated with this rare phenomenon, identifying SCLC as a risk factor for STLS is key. For a prompt diagnosis, close and regular monitoring of labs in these patients is recommended so that treatment, and perhaps even prophylaxis, can be delivered to patients with this rare carcinoma.
